# Bioinformatic-based approach for mutagenesis of plant immune Tm-2^2^ receptor to confer resistance against tomato brown rugose fruit virus (ToBRFV)

**DOI:** 10.3389/fpls.2022.984846

**Published:** 2022-09-30

**Authors:** Karla Rivera-Márquez, Leandro Alberto Núñez-Muñoz, Berenice Calderón-Pérez, Rodolfo De La Torre-Almaraz, Brenda Yazmín Vargas-Hernández, Roberto Ruiz-Medrano, Beatriz Xoconostle-Cázares

**Affiliations:** ^1^ Departamento de Biotecnología y Bioingeniería, Centro de Investigación y de Estudios Avanzados, Mexico City, Mexico; ^2^ Unidad de Biotecnología y Prototipos, Facultad de Estudios Superiores Iztacala, Universidad Nacional Autónoma de México, Tlalnepantla, Mexico, Mexico

**Keywords:** ToBRFV, resistance, Tm-2^2^, molecular docking, *in silico* mutagenesis

## Abstract

Nucleotide-binding leucine-rich repeat (NLR) plant immune receptors mediate the recognition and activation of defense signaling pathways in response to intra- and extracellular pathogens. Several NLR such as Tm-2 and Tm-2^2^ have been introgressed into commercial solanaceous varieties to confer protection against different tobamoviruses. Particularly, Tm-2^2^ was used during recent decades to confer resistance against tobacco mosaic virus, tomato mottle mosaic virus and tomato mosaic virus, which recognizes the viral movement protein (MP). However, tomato brown rugose fruit virus(ToBRFV), a novel tobamovirus, can avoid the protection conferred by Tm-2^2^ due to the presence of key substitutions in the MP. The aim of this work was to identify the key amino acid residues involved in the interaction between Tm-2^2^ and ToBRFV MP through bioinformatic analyses, and to identify potential Tm-2^2^ mutations that could generate greater binding affinity. *In silico* 3D structure prediction, molecular docking, and computational affinity methods were performed. We predicted that R350, H384 and K385 Tm-2^2^ residues are relevant for the interaction with MP, and two mutations (H384W and K385L) were identified as putative sites to increase the affinity of Tm-2^2^ to the MP with the potential elicitation of resistance against ToBRFV.

## Introduction

Solanaceae is one of the most economically important plant families extensively used in the food and pharmaceutical industries, with around 100 genera and 2,500 species currently recognized (Samuels, 2015). Solanaceae family includes plant species such as tomato (*Solanum lycopersicum*), potato (*S. tuberosum*), eggplant (*S. melongena*), pepper plants (*Capsicum* spp.), tobacco plants (*Nicotiana* spp.), among others (Ghatak et al., 2017). However, these crops are susceptible to viral infections, including members of the *Tobamovirus* genus, which have no insect vectors. Tobamoviruses are transmitted by plant sap and mechanical contact, although they are often seed-transmitted without the infection of the embryo. As virions are resilient, debris in the soil from infected tissue usually serves as source of infection (Kenyon et al., 2014; Koh et al., 2018). The *Tobamovirus* genus comprises more than 30 virus species, including *Tobacco mosaic virus*, *Tomato mosaic virus*, *Tomato mottled mosaic virus* and *Tomato brown rugose fruit virus* (ICTV, 2021).

As viral populations constantly evolve and adapt to agricultural environments, the need to develop control or mitigation strategies has increased (Elena et al., 2014). An active resistance response to viruses is mediated by plant disease resistance (R) genes, which encode for proteins containing nucleotide binding site (NBS) and C-terminal leucine-rich repeat (LRR) domains. Upon recognition of pathogen-derived elicitors, these NBS–LRR proteins initiate signaling pathways that generally lead to pathogen restriction (Bhattacharjee et al., 2009). In tomato varieties, R genes such as *Tm-1*, *Tm-2* and *Tm-2^2^* have been used to limit tomato mosaic virus (ToMV) and tobacco mosaic virus (TMV) infections. Tm-1 interferes with ToMV replication, while Tm-2 and Tm-2^2^ induce an elicitor triggered immunity (ETI) response. In some conditions, ETI can generate extreme resistance or hypersensitive response (HR), promote cell death following the interaction with the viral movement protein (MP) and prevent the spread of ToMV and TMV (Kobayashi et al., 2011; Zhang et al., 2013; Ishibashi and Ishikawa, 2014).

Tm-2^2^ gene was introgressed from *Solanum peruvianum* to *S. lycopersicum* providing a dominant, robust, and long-lasting resistance against TMV and ToMV strains (Lanfermeijer et al., 2003; Lanfermeijer et al., 2004). The introgressed gene segment comprised approximately half of chromosome 9 in a syntenic region part of pericentromeric heterochromatin (Lin et al., 2014; Van Rengs et al., 2022). Tm-2^2^ is composed by three domains: the coiled-coil domain (CC, including amino acids 1 to 141), NBS domain (residues 141-492) and LRR domain (residues 493-861) (Wang et al., 2020). All the domains of Tm-2^2^ are required to preserve its function and subcellular location in the plasma membrane as peripheric protein (Chen et al., 2017; Wang et al., 2020). W767 residue has a role in the activation of Tm-2^2^ (Kobayashi et al., 2011). Additionally, MP N-terminal end (1-187) is important in the Tm-2^2^ recognition (Weber Pfitzner, 1998; Weber et al., 2004; Chen et al., 2017); while S238 and K244 of the C-terminal end are related with overcoming of the resistance mediated by Tm-2^2^ (Weber et al., 1993). The small subunit of ribulose-1,5-bisphosphate carboxylase-oxygenase enzyme, the J-domain of MP-interacting protein 1, heat shock protein of 40 kDa (HSP40), HSP90 and the cochaperone SGT1 are positively involved in the process of extreme resistance against ToMV (Du et al., 2013; Zhao et al., 2013; Qian et al., 2018). Nevertheless, the molecular mechanism of Tm-2^2^-mediated viral resistance is largely unknown and the physical interactions between Tm-2^2^ and MP are not clearly understood.

The use of these R genes, especially Tm-2^2^, has decreased yield losses attributed to tobamoviruses in commercial plant varieties for many decades (Schouten et al., 2019). However, positive-stranded RNA viruses display an elevated mutation rate, which has led to the emergence of virus isolates able to break and evade the resistance conferred by Tm-1, Tm-2 and Tm-2^2^ (Meshi et al., 1988; Maayan et al., 2018). In fact, tomato brown rugose fruit virus (ToBRFV) which was first reported in Israel and Jordan between 2014 and 2015 (Salem et al., 2016; Luria et al., 2017) can break the resistance mediated by *Tm-1* and *Tm-2/Tm-2^2^* genes becoming a major agricultural threat (Hak and Spiegelman, 2021). The mechanism by which ToBRFV overcomes the resistance conferred by *Tm-2^2^* gene is unclear (Yan et al., 2021).

No effective measures have been identified to control ToBRFV infection, although some methods have been reported such as disinfectants against mechanical transmission (Chanda et al., 2021), thermal- and chemical-based disinfection treatments on ToBRFV-infected seeds (Davino et al., 2020; Samarah et al., 2021), and identification of tolerant genotypes of *Solanum* spp., including *S. chilense*, *S. habrochaites*, *S. lycopersicum*, *S. ochrantum*, *S. pennelli*, *S. peruvianum* and *S. pimpinellifolium* (Ashkenazi et al., 2018; Hamelink et al., 2019; Ykema et al., 2020; Jewehan et al., 2022a; Jewehan et al., 2022b; Kabas et al., 2022). Recently, simultaneous gene editing to knockout the four tobamovirus multiplication protein 1 (TOM-1) homologues in tomato was reported to confer strong resistance to ToBRFV (Ishikawa et al., 2022). With the absence of a commercial resistant cultivar, genetic resources for ToBRFV resistance are needed. Due to the high mutation rate, ToBRFV strains have appeared capable of breaking the natural resistance present in solanaceous species and even in edited plants, constituting a huge challenge to confer long-lasting resistance against ToBRFV (Jewehan et al., 2022c). Thus, the present work intended to determine key regions in the interaction between Tm-2^2^ and ToBRFV MP to identify potential changes in Tm-2^2^ that could increase the affinity between both proteins using bioinformatic tools. This could lead to a more efficient activation of ETI response in susceptible crops, such as most commercial and wild varieties of tomato, and to confer ToBRFV resistance.

## Material and methods

### Protein secondary and tertiary structure prediction

ToBRFV MP (accession number MK648157.1), ToMV MP (P69511), and TMV MP (NP_597748.1) secondary structure characterization was performed employing PDBSum software (http://www.ebi.ac.uk/thornton-srv/databases/pdbsum/Generate.html). Three-dimensional models of ToBRFV MP, ToMV MP, TMV MP and the resistance protein Tm-2^2^ (Q71BG9.1) were generated with trRosetta server (https://yanglab.nankai.edu.cn/trRosetta/) and AlphaFold version 2.0 (Jumper et al., 2021; Varadi et al., 2022). Subsequently, each set of models was validated using PROCHECK (Laskowski et al., 1993) and ERRAT (Colovos and Yeates, 1993) tools of the SAVES v6.0 platform (https://saves.mbi.ucla.edu/). The best predictions were selected based on the analysis of the Ramachandran plots and Z-scores calculated in the ProSA-web server (Wiederstein and Sippl, 2007). Selected models underwent a refinement using the GalaxyRefine software (https://galaxy.seoklab.org/cgi-bin/submit.cgi?type=REFINE) (Heo et al., 2013) and another validation was performed to select the most favorable model. Absolute quality estimates were calculated using the QMEAN server (http://swissmodel.expasy.org/qmean) (Benkert et al., 2011). Intramolecular contacts and energy profiles of the predicted structural models were calculated with ANOLEA (http://melolab.org/anolea/) (Melo and Feytmans, 1998). We also predicted the intrinsically unstructured regions of the ToBRFV, TMV and ToMV MP with the IUPRED2A software (https://iupred2a.elte.hu/) employing the long disorder prediction type (Mészáros et al., 2018)

### Protein overlay

The predicted 3D structures of Tm-2^2^, ToBRFV and TMV MP were used in protein-protein overlay analysis with UCSF Chimera software version 1.16 (Pettersen et al., 2004) to identify essential residues in the virus-host interaction of TMV MP and Tm-2^2^.

### Molecular docking

Molecular docking analyses were performed using the HADDOCK software version 2.2 (https://alcazar.science.uu.nl/services/HADDOCK2.2/haddockserver-easy.html). As this software generates the coupling considering the molecules as receptor and ligand (Domínguez et al., 2003; Van Zundert et al., 2016), reciprocal interactions (Tm-2^2^ with viral MP and vice versa) were analyzed. For Tm-2^2^, six different regions were covered (1-150, 151-300, 301-450, 451-600, 601-750 and 751-861) due to only 150 residues per simulation can be analyzed. For viral MP, the region between position 40 to 190 was selected including the active contact residues proposed by Yan et al. (2021). The determination of passive residues in both proteins was carried out using the default conditions of the software. The identification of interacting amino acids residues was carried out using the UCSF Chimera and PDBsum software (http://www.ebi.ac.uk/thornton-srv/databases/pdbsum/Generate.html). Root mean square deviation (RMSD) and Z-scores were considered to delimit interaction regions and to evaluate aminoacidic contacts to identify potential mutation candidates. The binding affinity (ΔG) and dissociation constant (K_d_) of each docking were calculated using the PRODIGY web server (https://wenmr.science.uu.nl/prodigy/) (Xue et al., 2016).

### *In silico* mutations

*In silico* mutations were performed with the MutaBind software version 2 (https://lilab.jysw.suda.edu.cn/research/mutabind2/), and binding affinity energies were evaluated (Zhang et al., 2020) for all possible mutations in each candidate, selecting the most favorable. Based on the primary structure, the stability of the Tm-2^2^ protein after single and multiple mutations was determined using the iStable software version 2 (http://ncblab.nchu.edu.tw/iStable2/) (Chen et al., 2020a). To determine the best *in silico* Tm-2^2^ protein variant to confer tolerance against ToBRFV, an affinity energy analysis was performed on the proposed mutants. We also employed PremPS software (Chen et al., 2020b) to calculate ΔΔG associated with each mutant and to visualize the non-covalent interactions between the chosen amino acids and the proposed mutants.

## Results

### ToBRFV MP, ToMV MP, TMV MP and Tm-2^2^ predicted secondary and tertiary structures

ToBRFV MP, TMV MP and ToMV MP shared similar secondary structures including 2 sheets, 1 β-hairpin, 3 β-bulges, 10 strands, 6 helices, 4 helix-helix interacts, 16 β-turns and 1 γ-turn (**Figures S1–S3**). ToBRFV MP showed an additional helix (**Figure S1**), ToMV MP showed 2 helix-helix interacts, 1 additional β-bulge, 1 additional β-turn and 1 additional γ-turn (**Figure S2**) while TMV MP showed 1 additional β-bulge, 3 β-turns and 3 γ-turns (**Figure S3**). Tm-2^2^ predicted model showed 6 sheets, 7 β/α/β units, 1 β- hairpin, 6 β-bulges, 32 strands, 36 helices, 43 helix-helix interacts, 63 β-turns and 5 γ-turns (**Figure S4**). We also identified intrinsically unstructured regions in the MP of ToBRFV, ToMV and TMV, located at the C-terminus (**Figure S5**). Several 3D structures were generated for ToBRFV, TMV and ToMV MP, and the protein coded by the *Tm-2^2^* resistance gene (**Figure S6 A, C, E, G**). The resulting models were further refined and validated to select the best structure prediction for each protein based on Ramachandran plots (**Figure S6 B, D, F, H**, **Table S1**) and energy profiles (**Figure S7**). Overall Quality Factor for non-bonded atomic interactions (with values >50 for high-quality models) and Z-Scores for MPs and Tm-2^2^ protein models also showed acceptable values for each of the models used subsequently in molecular docking experiments (**Table S2**).

Intramolecular contacts (**Figure S8**) and residue energy profiles (**Figure S9**) for ToBRFV MP and ToMV MP models were similar, while TMV MP model presented a lower number of average contacts and higher values of energy per residue. Residue energy profiles of Tm-2^2^ model showed negative values throughout its entire sequence; while MPs showed positive energy values close to the C-terminus region, like the results obtained for intrinsically unstructured regions.

### Identification of interacting amino acids in MP

For the Tm-2^2^ and ToBRFV/ToMV/TMV MP reciprocal interactions, molecular dockings were generated. Molecular docking results showed higher RMSD values in the interaction of Tm-2^2^ with ToBRFV (1.5) compared to ToMV and TMV (0.8), supporting the hypothesis of lower affinity of Tm-2^2^ to ToBRFV MP compared with other tobamoviruses (**Table S3**). The best docking model was selected based on the results of the HADDOCK server, showing RMSD 21.9 ± 0.1 kcal/mol (from the overall lowest-energy structure value), Van der Waals energy -17.5 ± 5.8 kcal/mol, electrostatic energy (-452.6 ± 52.0 kcal/mol), desolvation energy (20.3 ± 3.4 kcal/mol) and Z score (-2.0). The selected docking model showed 49 residues of Tm-2^2^ interacting with 56 residues of ToBRFV MP. Specific interactions between ToBRFV MP and Tm-2^2^ amino acids included 397 non-bonded contacts, 32 hydrogen bonds and 6 salt bridges. (**Supplementary File 1**). Most of the contacts for ToBRFV MP were located between residues 100 to 200 and for Tm-2^2^ contacts were grouped into 5 clusters: one close to residue 60, one close to residue 200, and 3 clusters between residues 300 and 400 (Fig S10).

**Figure 1:**
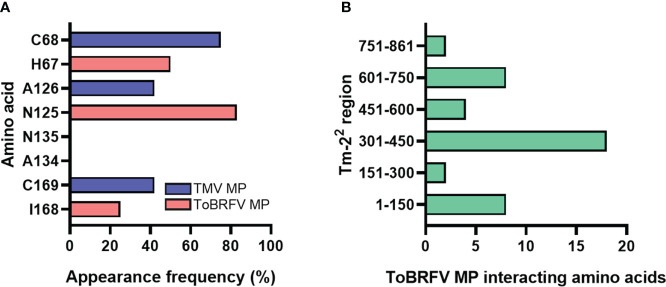
Identification of interacting amino acids between Tm-2^2^ and tobamoviral MP. **(A)** ToBRFV MP and TMV MP interacting residues in Tm-2^2^ regions. **(B)** Contacts generated by the PDBsum software between Tm-2^2^ and ToBRFV MP.

We also evaluated the affinity energies of substitutions H67C, N125A, K129Q, A134N, I147M, and I168N (Yan et al., 2021) in ToBRFV MP showing that most of them increase the affinity of ToBRFV MP upon interaction with Tm-2^2^ in reciprocal interactions (**Table S4**). Additionally, reciprocal interactions between Tm-2^2^ and ToBRFV MP revealed involvement of H67, N125, A134 and I168 amino acid residues as potentially important. To determine interacting residues of TMV MP, alignment and 3D structure overlay with ToBRFV MP were performed. In comparison, for Tm-2^2^ and TMV MP reciprocal interaction C68, A126, N135 and N169 were determined as key residues. These ToBRFV and TMV MP amino acids were present in the same conserved region. The contact amino acid relative frequency was then determined using these decoy residues (H67/C68, N125/A126, A134/N135 and I168/N169) for each of the Tm-2^2^ and ToBRFV/TMV MP reciprocal interactions, as shown in **Figure 1A**. ToBRFV MP residues N125, H67 and I168 showed appearance frequencies higher than 20%; while A134 did not appear in any docking calculation. A similar pattern was obtained with the homologous amino acids for TMV MP.

### Identification of essential interacting amino acids of Tm-2^2^

**Figure 2:**
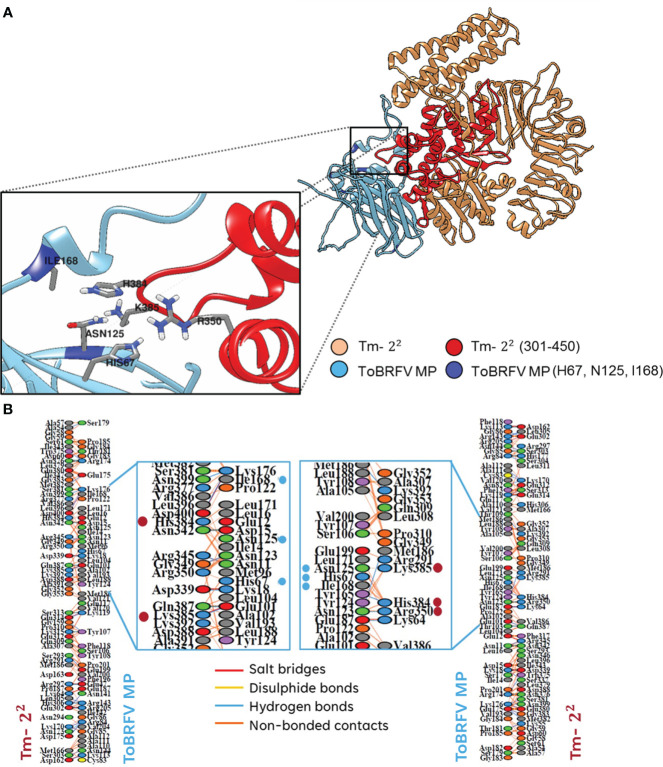
**(A)** Interaction between ToBRFV MP with Tm-2^2^. The interaction region of Tm-2^2^ with the indispensable amino acids of MP involved in resistance breaking is highlighted. **(B)** Interactions between the couplings of Tm-2^2^ with MP and vice versa. Essential residues in Tm-2^2^ are shown as red dots. The lines between residues indicate the type of identified contact: salt bridge in red, disulfide bridge in yellow, hydrogen bonds in blue and non-bonded interactions in orange. Color of the ovals connecting these lines corresponds to the type of amino acid: blue for positive residues (H, K, R), red for negative residues (D, E), green for neutral residues (S, T, N, Q), gray for aliphatic residues (A, V, L, I, M), purple for aromatic residues (F, Y, W), brown for proline and glycine; and yellow for cysteine.

On the other hand, to identify the amino acids that were predicted to establish molecular contact with H67, N125 and I168 of the ToBRFV MP with Tm-2^2^, several regions of this receptor were evaluated. The largest number of interacting residues in the docking were contained in the region between position 301-450 (**Figure 1B**). To validate the most favorable Tm-2^2^ region to interact with ToBRFV MP, the RMSD and Z score values were calculated from the molecular dockings predicted by the HADDOCK2.2 software (**Table 1**). Considering this region, amino acids that presented any type of molecular interaction with H67, N125 and I168 residues of ToBRFV MP were identified (**Figures 2A**, **B**). From these, R350, H384 and K385 residues were considered candidates for in *silico* mutation.

**Table 1:** Root mean square deviation (RMSD), Z-Score, binding affinity (ΔG) and dissociation constant (K_d_) for the reciprocal interaction between Tm-2^2^ and ToBRFV MP.

	Tm-2^2^ region					
	1 – 150	151 – 300	301 – 450	451 – 600	601 – 750	750 – 861	Interaction	
**RMSD**	1.0	0.6	1.5	1.9	11.3	11.7	Tm-2^2^ and MP
	2.2	2.8	1.1	17.3	0.8	2.5	MP and Tm-2^2^
**Z- Score**	-2.0	-1.8	-2.4	-2.1	-1.5	-2.1	Tm-2^2^ and MP
	-1.5	-2.0	-2.5	-1.9	-1.9	-1.6	MP and Tm-2^2^
**ΔG (kcal mol^-1^)**	-16.1	-21.0	-23.7	-19.8	-20.5	-20.0	Tm-2^2^ and MP
	-18.1	-17.1	-24.6	-19.9	-18.4	-16.4	MP and Tm-2^2^
**K_d_ (M at 25°C)**	1.3×10^-12^	3.9×10^-16^	4.5×10^-18^	2.9×10^-15^	9.8×10^-16^	2.0×10^-15^	Tm-2^2^ and MP
	5.3×10^-14^	2.8×10^-13^	9.3×10^-19^	2.4×10^-15^	3.4×10^-14^	9.2×10^-13^	MP and Tm-2^2^

### *In silico* mutations

Possible mutations were evaluated by testing R350, H384 and K385 with 19 different amino acid substitutions at each position. The free folding energy (ΔΔG) values and non-covalent interactions demonstrated the affinity of the reciprocal interaction between the Tm-2^2^ and ToBRFV MP with amino acid substitutions (**Table 2** and **Figure S11**). Mutation-free position (H384 and K385), two single mutations (H384W and K385L), and a double substitution (H384W with K385L) were analyzed.

**Table 2:** Affinity energy in Tm-2^2^ mutants.

	Refolding free energy (ΔΔG, kcal mol^-1^)	
Tm-2^2^	Tm-2^2^ – ToBRFV MP	ToBRFV MP – Tm-2^2^	
H384 K385	0.03	1.23	
H384W	-0.98	-0.37	
K385L	-0.38	-0.58	
H384W K385L	-1.1	-1.3	

**Figure 3:**
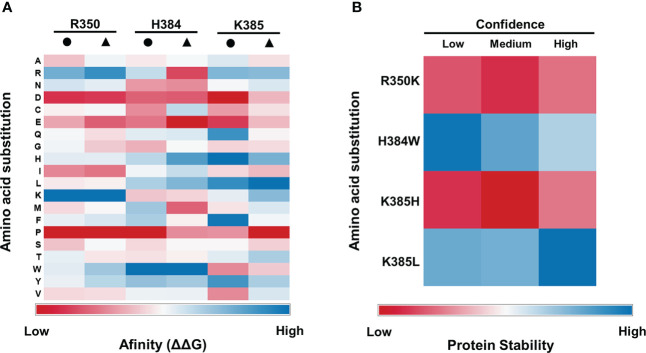
Affinity plots for potential mutations in Tm-2^2^ for ToBRFV resistance. **(A)** Affinity of folding free energy (ΔΔG) for all possible mutations at residues R350, H384 and K385. **(B)** Affinity stability of the primary structure of the Tm-2^2^ protein, for possible mutation candidates R350K, H384W, K385H and K385L. Black circles represent interaction affinity columns using Tm-2^2^ as rigid molecule and ToBRFV MP as flexible molecule. Black triangles represent interaction affinity columns using ToBRFV MP as rigid molecule and Tm-2^2^ as flexible molecule.

Based on the folding free energies obtained by the MutaBind2 software, an affinity plot (ΔΔG) was constructed (**Figure 3A**). The visual scale ranges from dark blue which indicates a maximum of free energy folding that leads to a spontaneous reaction, to red, in which the free energy is minimal and thus the interaction would hardly take place. In contrast, white color indicates an insignificant energy change. Virtual mutations that were able to increase the interaction affinity between Tm-2^2^ and ToBRFV MP were identified. For the R350 residue, the substitution of arginine with lysine (R350K) was favorable, unlike the substitution for isoleucine or valine that slightly decreased the affinity of the coupling; in contrast, substitution with tryptophan or tyrosine generated only a small increase in affinity. For H384, the substitution histidine for tryptophan (H384W) showed a high increase in the interaction affinity, compared to substitutions with asparagine and lysine. Similarly, substitutions with glutamine, isoleucine, leucine, phenylalanine, or tyrosine generated very weak enhancement for this coupling. Finally, the candidate K385 presented an increase in relevant affinity when substituted with histidine (K385H) in the interaction of Tm-2^2^ with MP, and leucine (K385L) in the reciprocal interaction. For this candidate, substitution of lysine with cysteine, glycine, isoleucine or tryptophan resulted in a decrease of affinity in both interactions, whereas the use of arginine, asparagine, glutamine, phenylalanine, threonine or tyrosine would only generate a slight increase in affinity. Finally, it was observed that for all the candidates the substitution by aspartic acid, glutamic acid or proline, were highly detrimental due to an excessive decrease in affinity.

In addition, changes in stability of mutations that were predicted to increase the interaction affinity between Tm-2^2^ and ToBRFV MP were evaluated using the iStable2 software (**Figure 3B**). The visual scale ranges from red to blue based on the effect on protein primary structure stability. For K385, both K385H and K385L substitutions were considered to determine whether both were stable. ΔΔG calculated with the PremPS software showed values of -0.86 (R350K), -0.73 (H384W), -0.78 (K385H) and -0.50 (K385L), suggesting that all mutations were stable.

## Discussion

Several economic damages have been reported on tomato and pepper open field and greenhouse production caused by ToBRFV infection (Cambrón-Crisantos et al., 2019; Panno et al., 2020). This virus overcomes resistance of the *Tm-2/Tm-2^2^* (and *Tm-1*) R genes in tomato (Luria et al., 2017). As no effective measures to control infection or a commercial resistant cultivar are available, genetic resources for ToBRFV resistance are needed. In the present work, key residues in the interaction of Tm-2^2^ with ToBRFV MP were determined through bioinformatic analysis and potential amino acid changes in the plant receptor were identified. We identified intrinsically unstructured regions at the C-terminus of ToBRFV MP, ToMV MP and TMV MP. Intrinsically unstructured regions have been previously identified at the C- terminus of South African cassava mosaic virus MP (Nankoo et al., 2022) and at the N-terminus of barley stripe mosaic virus MP (TGB1) (Semashko et al., 2012; Mishra et al., 2020). The structural and functional differences related with the location of these intrinsically disordered regions in MPs remain unclear.

Fist, from an extensive analysis of all predicted tertiary structures, the final models for ToBRFV MP, TMV MP, ToMV MP and Tm-2^2^ were selected. More than 90% of the residues of these models were present in the most favorable region of the Ramachandran graphs, confirming that they are highly reliable (Laskowski et al., 1993). Based on these models, it was possible to predict the molecular dockings in order to determine key residues in the virus-host interaction with ToBRFV, using TMV as a reference.

MP was selected for the analysis of virus-host interaction in the present study because it was previously reported that replicase and MP domain residues from ToBRFV and TMV overcome resistance against Tm-2^2^ (Maayan et al., 2018). We evaluated the predicted interaction of Tm-2^2^ and viral MPs in terms of affinity energy. We found that ToMV MP and TMV MP have higher affinity with Tm-2^2^ compared to ToBRFV MP. This supported the hypothesis of a decrease in affinity of ToBRFV MP with Tm-2^2^. A specific region of ToBRFV MP comprised between 60-186 is indispensable for virus-host interaction because it is involved in breaking the resistance conferred by Tm-2^2^ in *S. lycopersicum* (Yan et al., 2021). To select interacting residues, the participation of 12 essential ToBRFV MP amino acids that allow evading resistance against Tm-2^2^ was considered (Maayan et al., 2018). Furthermore, in a recent study, six ToBRFV MP mutants (in H67, N125, K129, A134, I147 and I168 positions) were identified to break the resistance conferred by Tm-2^2^ in *S. lycopersicum* (Yan et al., 2021). Considering these amino acids residues of ToBRFV MP, we calculated the affinity energies for each mutant: H67C, N125A, K129Q, A134N, I147M and I168N. Interestingly, H67C, N125A and I168N residues were located at the interface region between Tm-2^2^ and ToBRFV MP. Furthermore, H67C, A134N, I147M and I168N mutants showed increased affinity energies upon interaction with Tm-2^2^. K129Q may be involved in molecular mechanisms such as recognition between Tm-2^2^ and MP or interactions with components of the cellular response to pathogens or in the Tm-2^2^ activation.

As mentioned, four amino acids coincided in both previous studies (Maayan et al., 2018; Yan et al., 2021), which were used as the basis for this work. Our alignment and protein-protein overlay analysis indicated H67/C68, N125/A126, A134/N135 and I168/N169 as the amino acids of ToBRFV/TMV MP to be used in all molecular docking analyses. For the interaction of Tm-2^2^ with ToBRFV MP (**Figure 2**), the participation of only three amino acids was observed. N125 showed a relative frequency higher than 80%, unlike H67 and I168 which had a relative frequency lower than 60%. In the case of Tm-2^2^ and TMV MP, the amino acid with the greatest participation was C68, with a value above 60%. A126 and N169 reached values between 40 and 50% of relative frequency. For both MPs, residues A134 and N135 did not show any predicted interaction with Tm-2^2^.

Subsequently, Tm-2^2^ was subdivided into regions to identify the residues involved in the reciprocal interaction with both MPs. Viral MP residues H67/C68, N125/A126 and I168/N169 were used to identify interactions in regions 1 to 150, 151 to 300, 301 to 450, 451 to 600, 601 to 750 and 751 to 861 of Tm -2^2^ (**Figure 3**). Considering the values of RMSD and Z score (**Table 1**) the highest participation of residues and the best predictions of reciprocal interactions of Tm-2^2^ with MP were those spanned the region 301 to 450 of Tm-2^2^. It should be noted that the RMSD values are not predictive and only allow to identify the best generated model. On the other hand, the Z score refers to statistical values that represent how many standard deviations a given score differs from the average score. As a point of comparison, RMSD values lower than 2.0 predict better interaction models, and the more negative the Z score value, the more frequent the interaction (Dominguez et al., 2003; Van Zundert et al., 2016). Likewise to the selected region (301-450) based on the values generated by the molecular docking software, a previous study described the interaction of MP with NBS domains present in Tm-2^2^ and reported that these domains were found between residues 239 to 492 (Wang et al., 2020).

Once the Tm-2^2^ region was delimited, all the residues that showed molecular contact with H67, N125 and I168 in the ToBRFV MP were identified. Two types of interactions were identified. Hydrogen bonds in H67 and I168 with residue H384 of Tm-2^2^ were recognized. While non-bonded interactions, which involves weak contacts such as electrostatic interactions and Van der Waals forces (Laskowski, 2001), were identified in Tm-2^2^ residue R350 with H67, H384 with H67, N125 and I168, and K385 with N125, thus rendering them as potential alternatives of mutation candidates. The affinity plot (**Figure 3A**) was used to identify the most favorable mutation for each Tm-2^2^ residue. For R350 residue, the substitution of arginine with lysine (R350K) was favorable. For H384, the substitution of histidine for tryptophan (H384W) showed a high increase in the interaction affinity. Finally, K385 residue presented an increase in relevant affinity when substituted with histidine (K385H) in the interaction of Tm-2^2^ with MP, and leucine (K385L) in the reciprocal interaction.

For these four mutations, a stability analysis of Tm-2^2^ was performed to identify whether the substitutions could affect protein function (**Figure 3B**). This stability depends on the ΔΔG value, which reflects physical and biological properties of the protein, and it is defined based on Gibbs free energy change of the substituted protein relative to the non-modified molecule (Chen et al., 2020a). The stability decreased for the R350K and K385H mutations, so these substitutions were discarded. In contrast substitutions H384W and K385L showed an increase in stability, being the most favorable to confer resistance in plants. Energy change values that accompanied individual mutations shown in **Table 2** helped to identify those that may not affect Tm-2^2^ protein function. Affinity energies can be classified as highly destabilizing (ΔΔ*G* ≥ 1.0 kcal mol^-1^) or highly stabilizing (ΔΔ*G* ≤ -1.0 kcal mol^-1^) (Chen et al., 2020a). The affinity in the reciprocal interactions of Tm-2^2^ with native ToBRFV MP presented positive refolding free energy values (0.03 and 1.23). The analyzed substitutions (H384W, K385L and doble substitution H384W, K385L) showed a decrease in ΔΔG values, meaning that these would occur spontaneously and therefore the recognition of MP by Tm-2^2^ would be energetically favorable. The analysis of these mutations showed that the H384W substitution could be the most favorable because it had the lowest value (-0.98) compared to K385L (-0.38). However, the MP coupling with Tm-2^2^ harboring the H384W and K385L substitutions presented similar values. Finally, the best *in silico* substitution was the double mutation. It is important to consider that ToBRFV is an RNA virus, and it has a high mutation rate. A single infectious transcript can give rise to 100,000 viral copies in about 10 hours (Moya et al., 2004). Thus, resistance conferred by a Tm-2^2^ double substitution could be more difficult to overcome by ToBRFV, at least when compared to a single substitution.

Bioinformatic tools can be very useful to guide novel strategies for crop improvement. *In silico* 3D protein structure, molecular interactions, as well as proposed mutations were obtained to help explaining how this virus overcomes resistance of tomato cultivars harboring the *Tm-2^2^* gene and to identify essential residues involved in the evasion of resistance conferred by this genotype. Most of the data obtained in this study coincided for the prediction of key amino acids in MP interacting with Tm- 2^2^ (Wang et al., 2020). However, proposed mutations in this analysis must be corroborated experimentally. One approach to achieve this could be gene editing, already described for these crops for other gene targets.

One limitation of this study relies on the assumption that resistance to ToBRFV is based only in two amino acid substitutions in Tm-2^2^. However, positive sense ssRNA viruses such as tobamoviruses have high mutation rates and the emergence of resistant viral quasispecies as result of selection pressure is a common phenomenon. Furthermore, biological verification is needed to confirm that proposed mutations in Tm-2^2^ can trigger an ETI response leading to ToBRFV MP recognition and subsequently a HR response. The combination of our strategy with others such as gene editing could be a robust arsenal to generate long-lasting resistance against ToBRFV. In addition, to our knowledge this study constitutes the first example of use of structural bioinformatics tools for prediction and generation of Tm-2^2^ mutants to generate resistance against ToBRFV in tomato.

## Data availability statement

The original contributions presented in the study are included in the article/**Supplementary Material**. Further inquiries can be directed to the corresponding author.

## Author contributions

KR-M and LN-M made almost all bioinformatic predictions, LN-M and BX-C conceived the idea, KR-M, LN-M, BC-P and BV-H helped writing the manuscript draft, RT-A, RR-M and BX-C supervised the study and obtained the funding. All authors contributed to the article and approved the submitted version.

## Acknowledgments

The authors thank to Erika Isabella García-Mendoza, José Juan Durán-Zarate, and Hilda Cristina Ruiz-Serrano for their valuable technical support. KR-M specially thanks to Leticia Aguilar-Doroteo and Ana Magdiel Iturbide-Hernández for all the valuable advice and recommendations. LAN-M thanks to Posgrado de Ciencias Biológicas, Universidad Nacional Autónoma de México (UNAM).

## Conflict of interest

The authors declare that the research was conducted in the absence of any commercial or financial relationships that could be construed as a potential conflict of interest.

## Publisher’s note

All claims expressed in this article are solely those of the authors and do not necessarily represent those of their affiliated organizations, or those of the publisher, the editors and the reviewers. Any product that may be evaluated in this article, or claim that may be made by its manufacturer, is not guaranteed or endorsed by the publisher.
