# Supplementary material for: Bioinformatic-based approach for mutagenesis of plant immune Tm-22 receptor to confer resistance against tomato brown rugose fruit virus (ToBRFV)
Source: Front Plant Sci. 2022 Sep 30;13:984846. doi: 10.3389/fpls.2022.984846 (PMC9562835; doi:10.3389/fpls.2022.984846)
Supplement: Supplementary file 1 [file DataSheet_1.docx]

Supplementary Material

Supplementary Figures


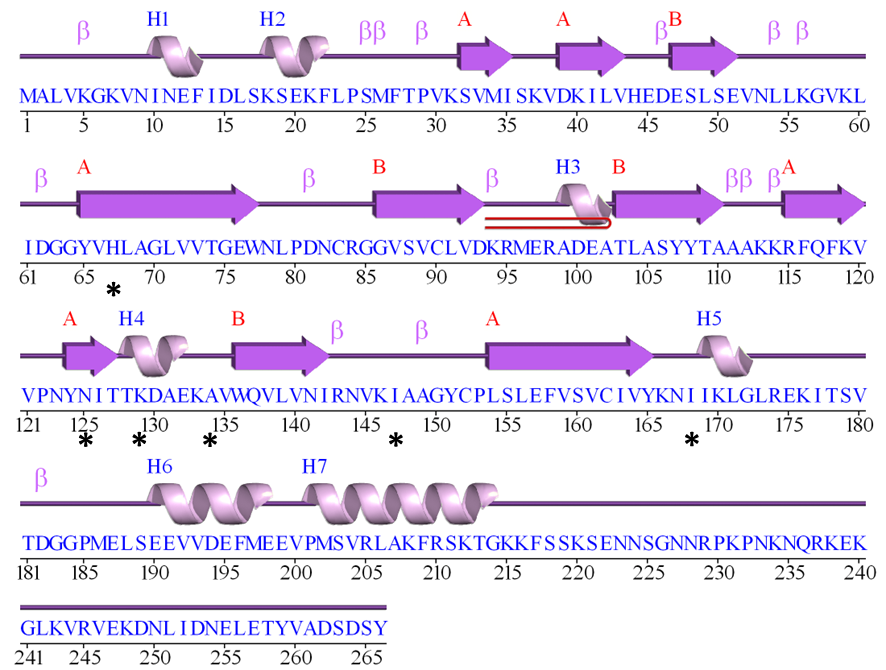


**Figure S1.** Predicted secondary structure of ToBRFV MP (accession number MK648157.1). Black asterisks indicate potential contact amino acids according to Yan et al., 2021. Helices (spirals) are identified as H1-H7, strands (arrows) are identified as A or B, depending on the type. Motifs can be β-turns (β), γ-turns (γ) and β-hairpin (red loop).


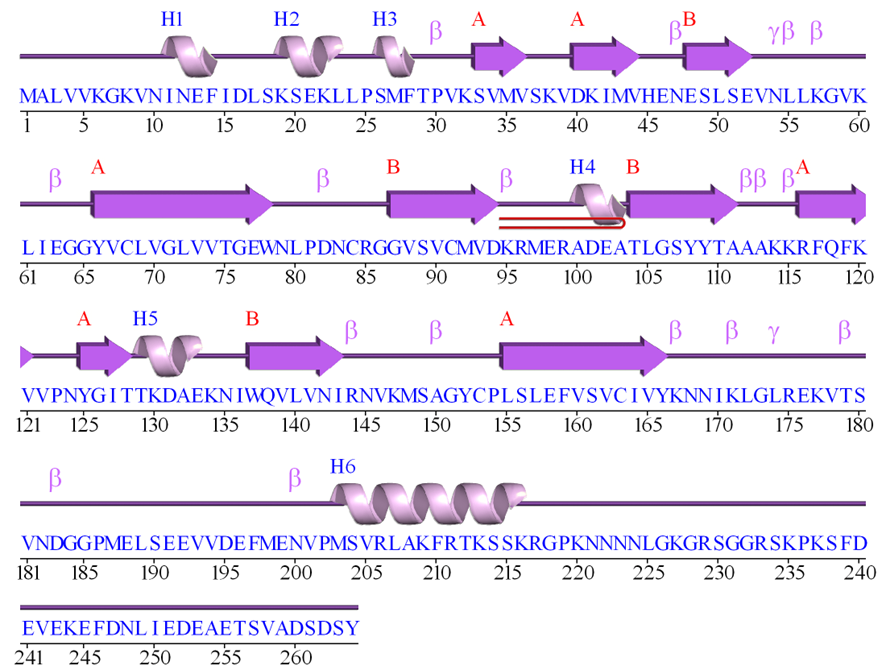


**Figure S2.** Predicted secondary structure of ToMV MP (accession number P69511). Helices (spirals) are identified as H1-H6, strands (arrows) are identified as A or B, depending on the type. Motifs can be β-turns (β), γ-turns (γ) and β-hairpin (red loop).

**
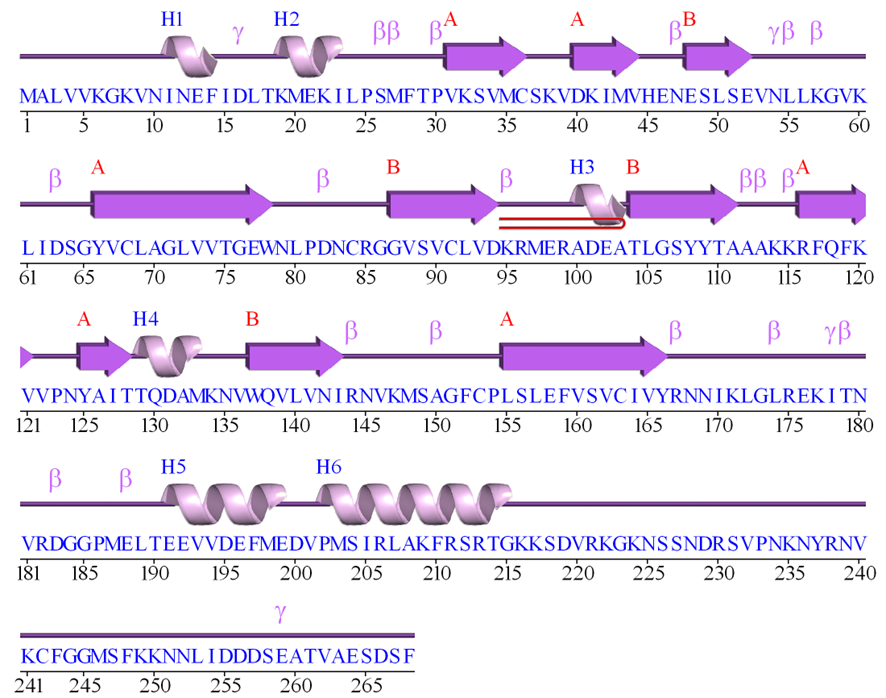
**

**Figure S3.** Predicted secondary structure of TMV MP (accession number NP_597748.1). Helices (spirals) are identified as H1-H6, strands (arrows) are identified as A or B, depending on the type. Motifs can be β-turns (β), γ-turns (γ) and β-hairpin (red loop).


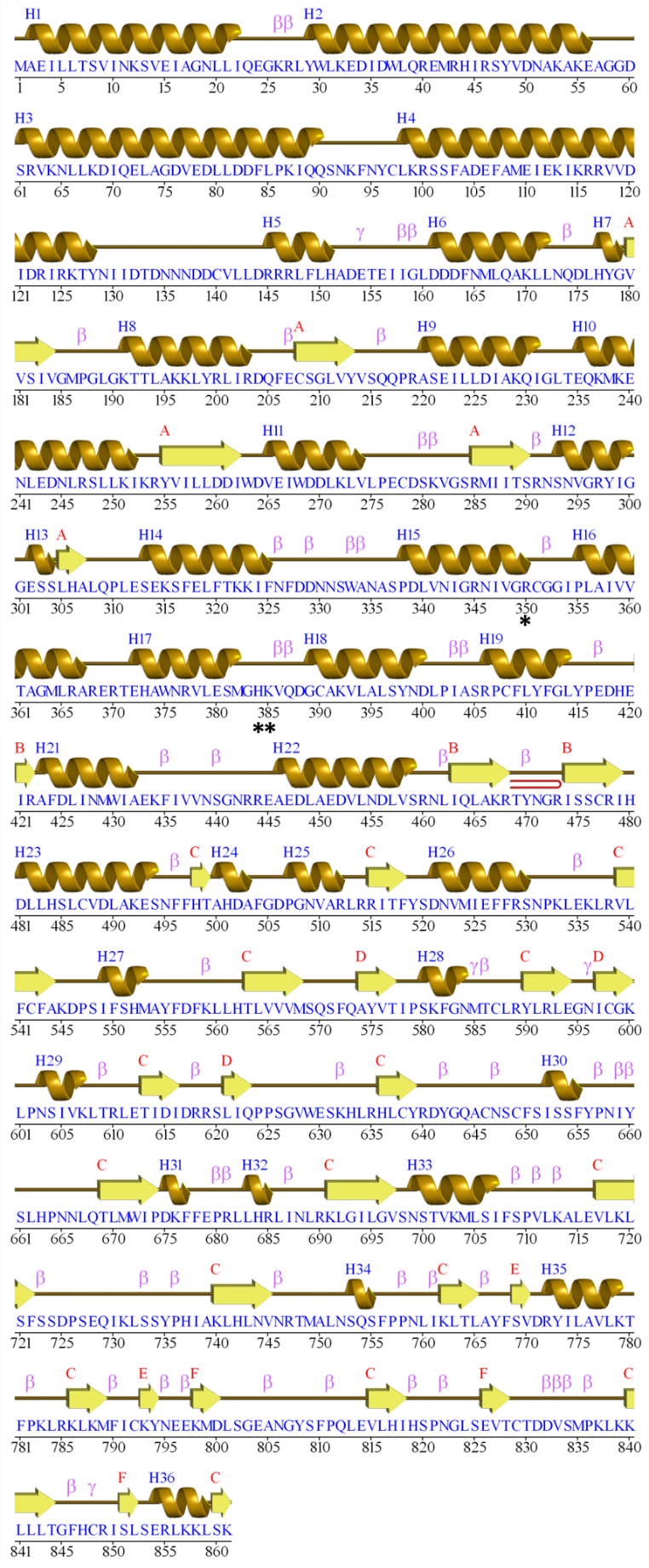


**Figure S4.** Predicted secondary structure of Tm-2^2^ (accession number Q71BG9.1). Black asterisks indicate potential contact amino acids predicted in this study. Helices (spirals) are identified as H1-H36, strands (arrows) are identified as A to F, depending on the type. Motifs can be β-turns (β), γ-turns (γ) and β-hairpin (red loop).


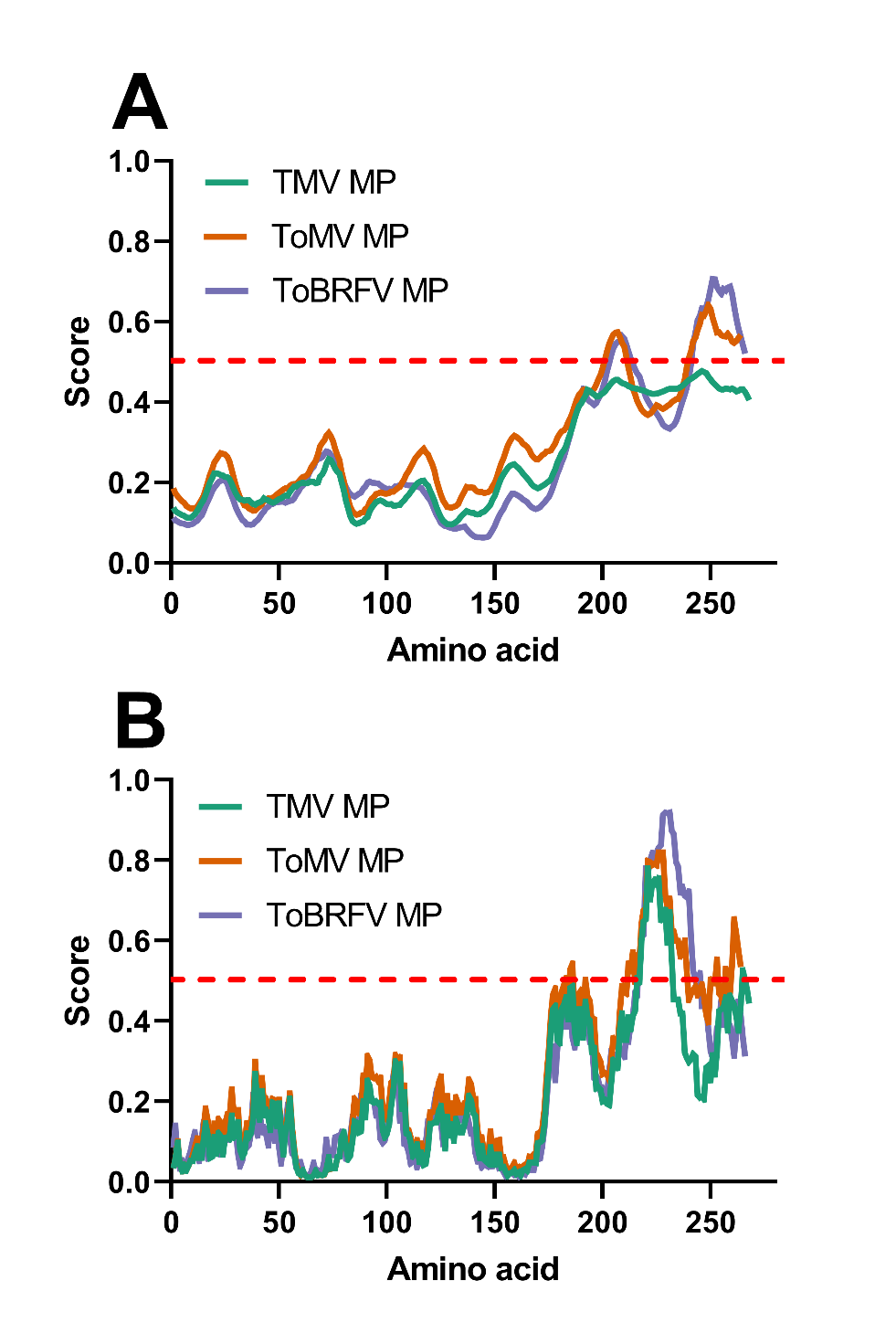


**Figure S5.** Prediction of intrinsically unstructured regions of ToBRFV MP, ToMV MP and TMV MP using Anchor (A) and IUPRED (B) scores. Red dashed line shows the threshold score. Values above the threshold are considered as intrinsically unstructured regions.


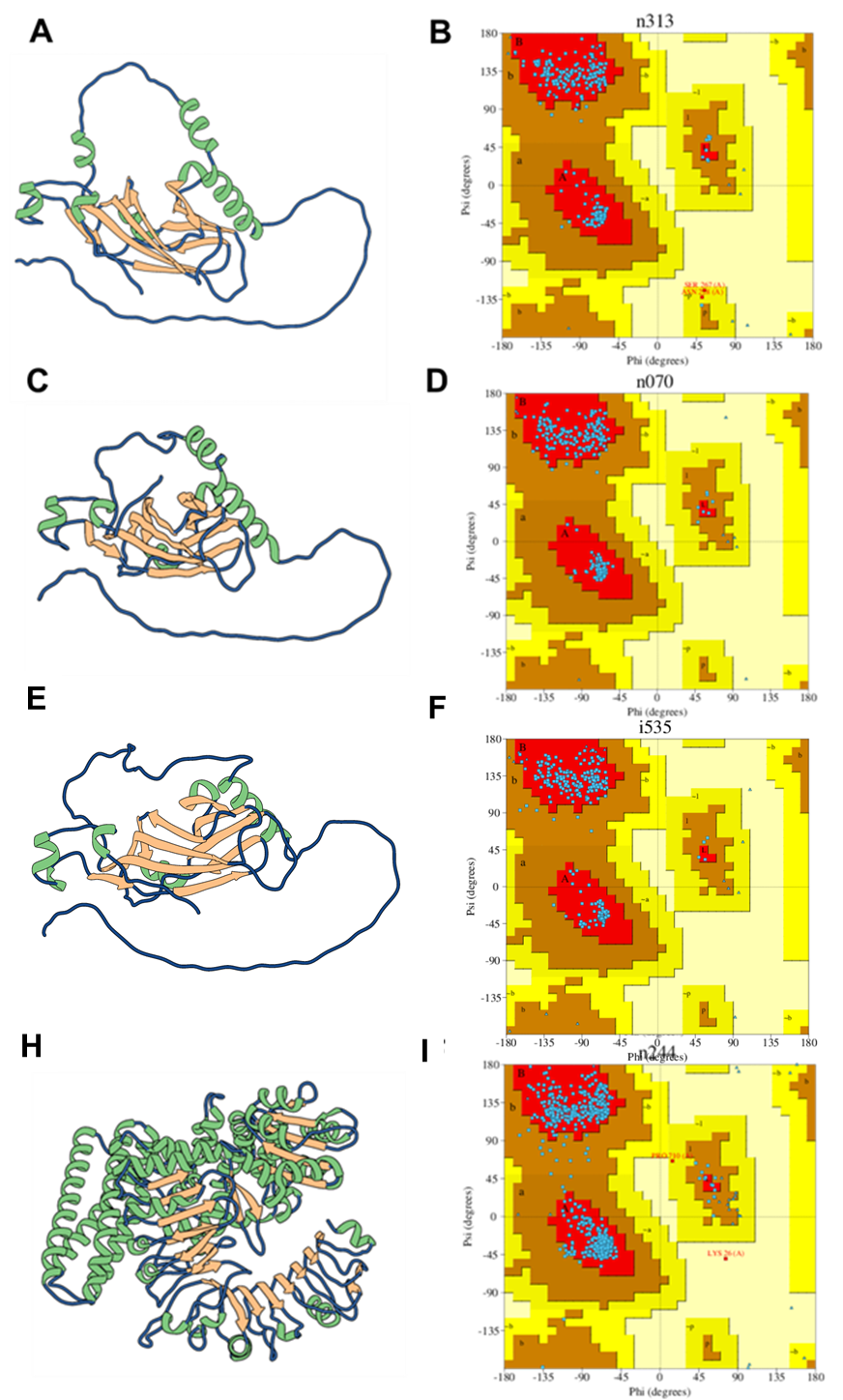


Figure S6. Predicted 3D structures and model validation of ToBRFV MP, TMV MP, ToMV MP and Tm-2^2^. Three-dimensional model prediction of ToBRFV MP (A), TMV MP (C), ToMV MP (E) and Tm-2^2^ (G). Ramachandran plot for the ToBRFV MP (B), TMV MP (D), ToMV (F) and Tm-2^2^ (H) models. Panels A, C, E and G show helices colored in green, coils in blue and strands in beige.


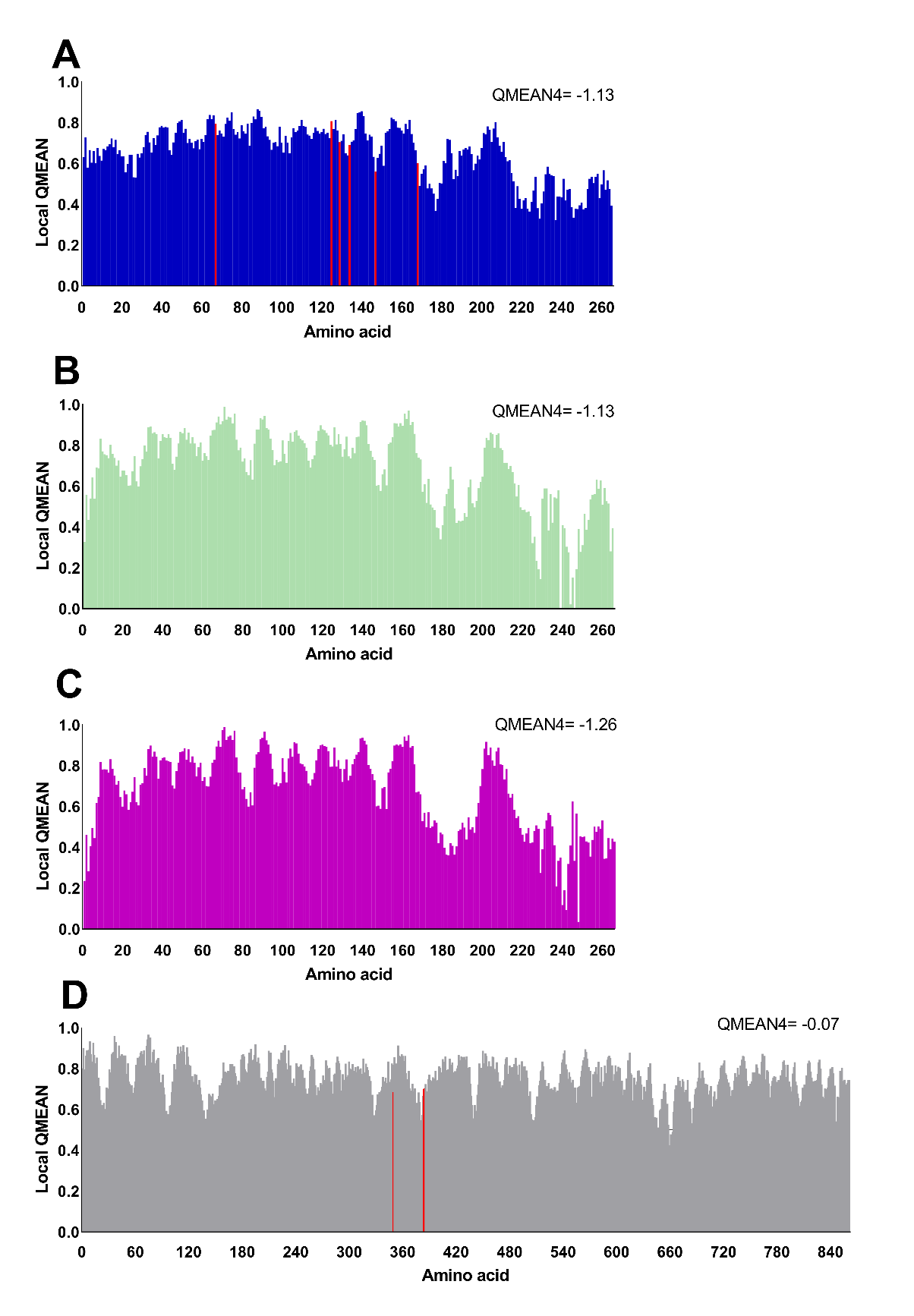


**Figure S7.** Absolute quality estimates for MP and Tm-2^2^. Local (QMEAN) and global (QMEAN4) values are showed for ToBRFV MP (A), ToMV MP (B), TMV MP (C) and Tm-2^2^ (D) predicted models. Red bars represent the local quality values obtained for residues considered relevant in the interaction ToBRFV and Tm-2^2^.


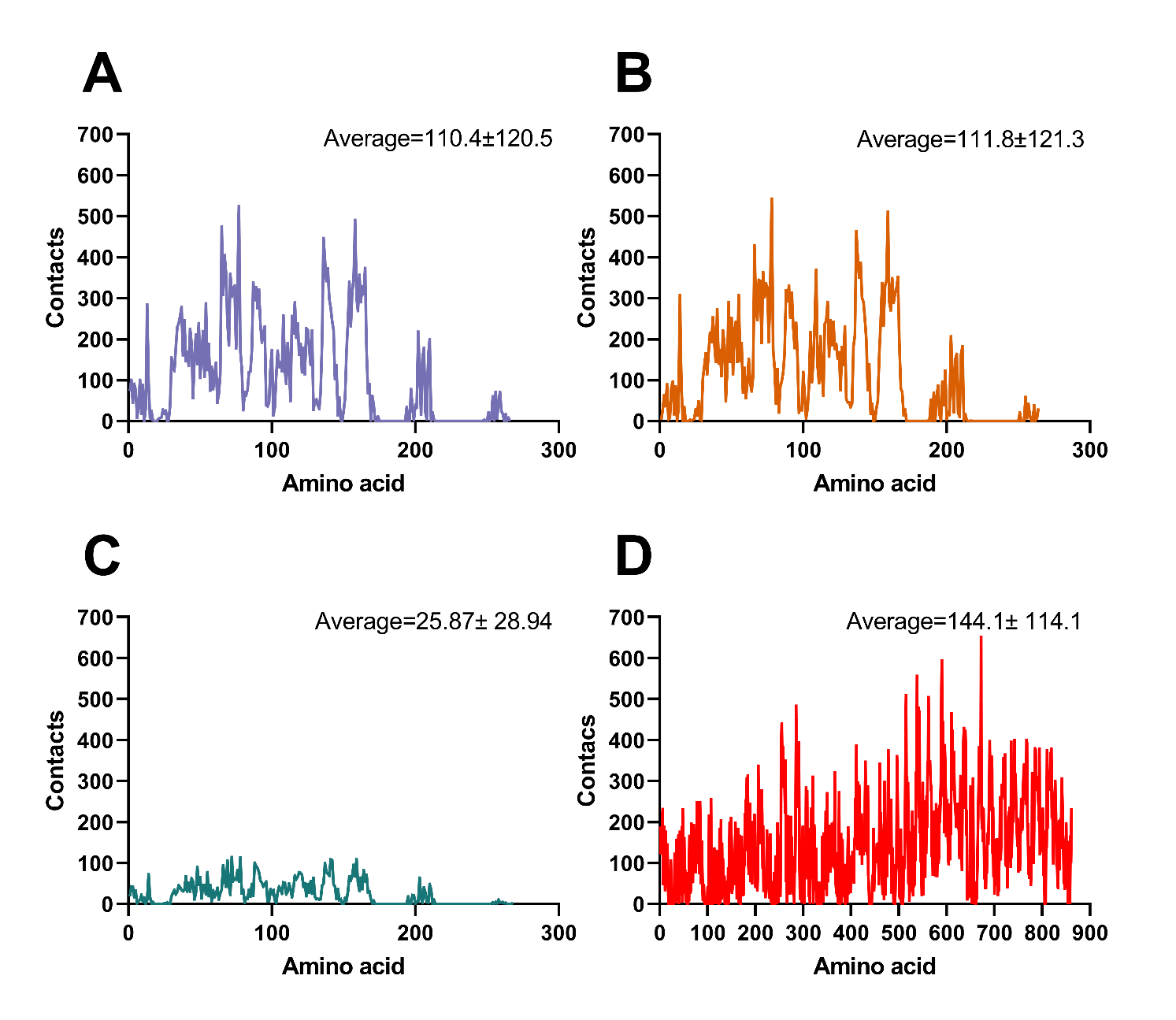


**Figure S8.** Intramolecular contact profile for the predicted structures of ToBRFV MP (A), ToMV MP (B), TMV MP (C), and Tm-2^2^ (D). The average contact value is shown in the upper right section of each panel.


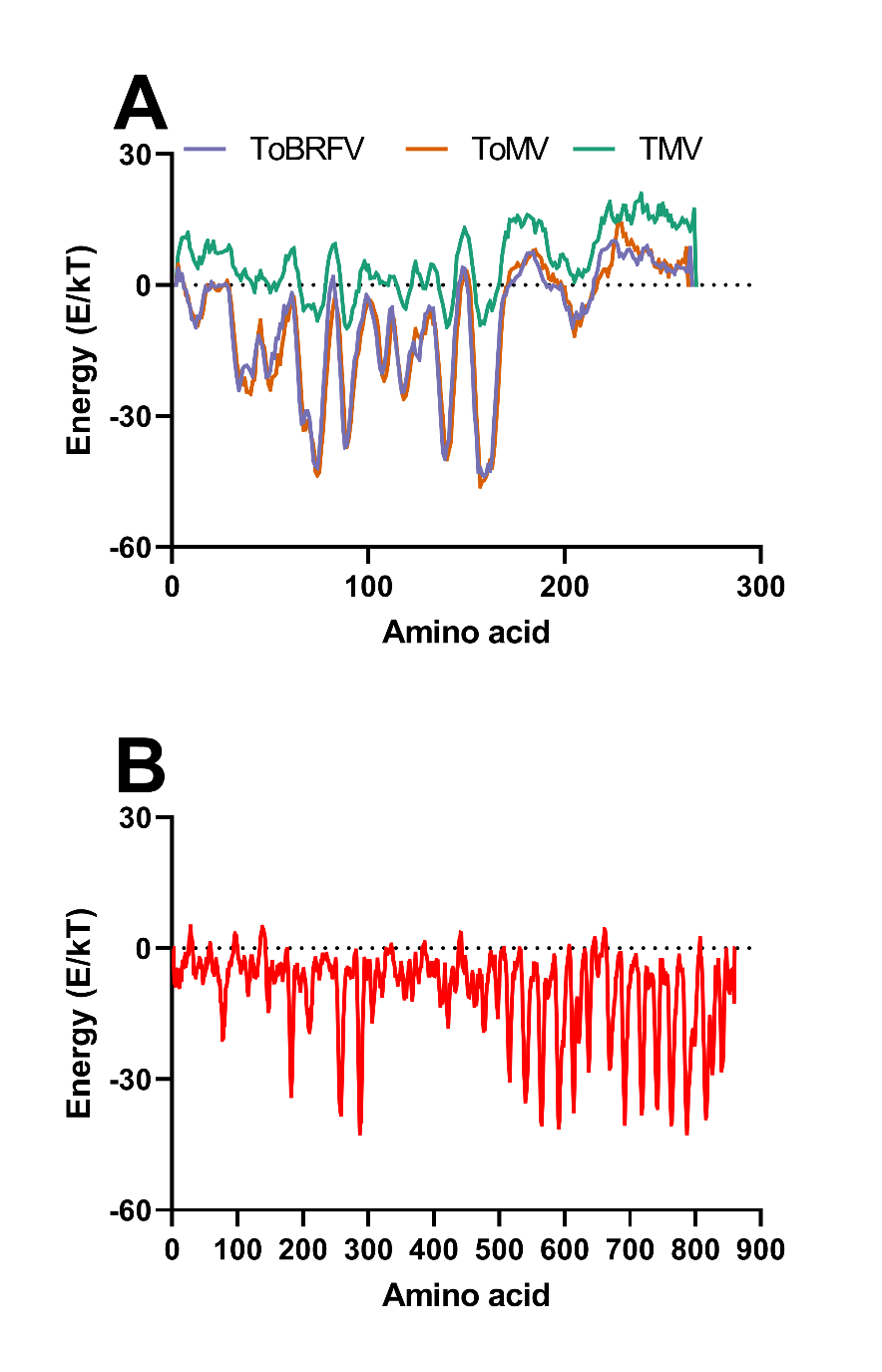


**Figure S9.** Energy profiles for each residue of the predicted models of tobamoviral MPs (A) and Tm-2^2^ (B).


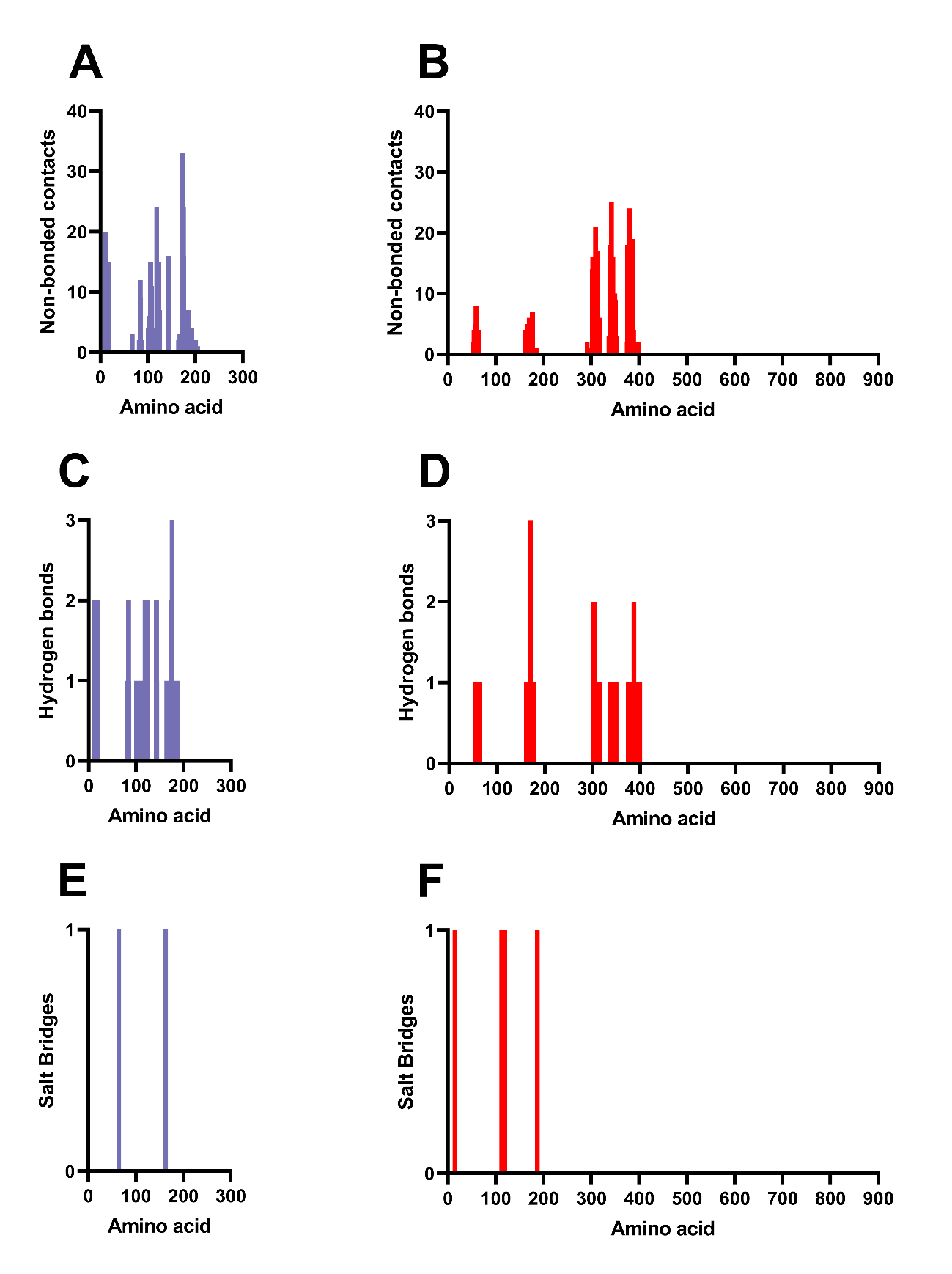


**Figure S10.** Atom-atom interactions across ToBRFV MP and Tm-2^2^ interacting interface. Non-bonded contacts for ToBRFV MP (A) and Tm-2^2^(B). Hydrogen bonds for ToBRFV MP (C) and Tm-2^2^ (D). Salt bridges for ToBRFV MP (E) and Tm-2^2^ (F).


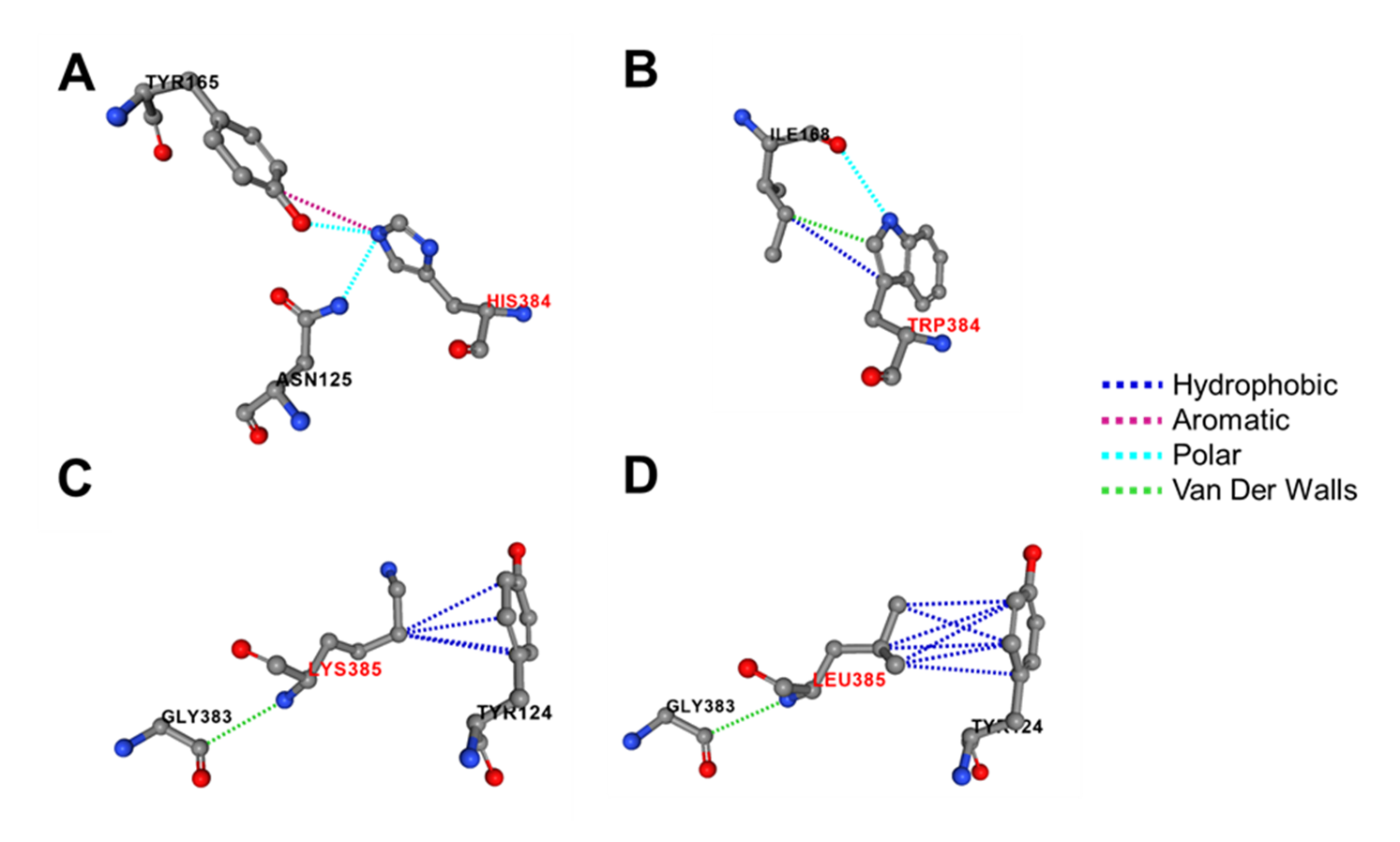


**Figure S11.** Non-covalent interactions of amino acids proposed for mutation in Tm-2^2^ and their interaction with ToBRFV MP amino acids. A) Tm-2^2^ wild type H384 interactions with ToBRFV MP residues B) Tm-2^2^ mutant H384K interactions with ToBRFV MP residues. C) Tm-2^2^ wild type K385 interactions with ToBRFV MP residues. D) Tm-2^2^ mutant K385L interactions with ToBRFV MP residues.

Supplementary Tables

**Table S1.** Ramachandran graph values for ToBRFV MP, ToMV MP, TMV MP and Tm-2^2^ predicted models with trRossetta and Alphafold2. The selected models are highlighted in light blue.

| **Protein target** | **Workflow** | **Ramachandran plot amino acid distribution** | | | | |
| --- | --- | --- | --- | --- | --- | --- |
|  |  | **Most favored regions [A,B,L] (%)** | **Additional allowed regions [a,b,l,p] (%)** | **Generously allowed regions [~a,~b,~l,~p] (%)** | **Disallowed regions (%)** |  |
| ToBRFV MP | trRosseta Unrefined | 92.1 | 7.1 | 0.8 | 0.0 |  |
|  | trRosseta Refined | 95.6 | 4.0 | 0.4 | 0.0 |  |
|  | Alphafold2 Unrefined | 68.3 | 17.5 | 9.6 | 4.6 |  |
|  | Alphafold2 Refined | 95.8 | 4.2 | 0.0 | 0.0 |  |
| TMV MP | trRosseta Unrefined | 92.1 | 7.4 | 0.0 | 0.4 |  |
|  | trRosseta Refined | 94.2 | 5.4 | 0.0 | 0.4 |  |
|  | Alphafold2 Unrefined | 67.3 | 22.3 | 7.0 | 3.3 |  |
|  | Alphafold2 Refined | 95.0 | 4.1 | 0.8 | 0.0 |  |
| ToMV MP | trRosseta Unrefined | 92.1 | 7.1 | 0.8 | 0.0 |  |
|  | trRosseta Refined | 94.4 | 5.1 | 0.4 | 0.0 |  |
|  | Alphafold2 Unrefined | 66.7 | 17.9 | 10.7 | 4.7 |  |
|  | Alphafold2 Refined | 95.7 | 4.3 | 0.0 | 0.0 |  |
| Tm-2^2^ | trRosseta Unrefined | 86.6 | 12.3 | 0.6 | 0.5 |  |
|  | trRosseta Refined | 91.1 | 8.1 | 0.4 | 0.4 |  |
|  | Alphafold2 Unrefined | 88.9 | 9.0 | 1.6 | 0.5 |  |
|  | Alphafold2 Refined | 95.2 | 4.7 | 0.0 | 0.1 |  |

**Table S2.** Overall Quality Factor and Z-Scores for MPs and Tm-2^2^ protein models

| **Protein model** | **Overall Quality Factor** | **Z-Score** |
| --- | --- | --- |
| ToBRFV MP | 91.8989 | -6.14 |
| ToMV MP | 98.2558 | -6.82 |
| TMV MP | 96.1957 | -6.76 |
| Tm-2^2^ | 94.4645 | -13.34 |

**Table S3**. Root mean square deviation (RMSD), Z-Score and binding affinity (ΔG) values generated in the interaction between Tm-2^2^ and ToBRFV MP, ToMV MP and TMV MP.

| **Viral species** | **RMSD** | **Z-Score** | **ΔG (kcal mol^-1^)** |
| --- | --- | --- | --- |
| ToBRFV | 1.5 | -1.8 | -18.8 |
| ToMV | 0.8 | -2.0 | -23.5 |
| TMV | 0.8 | -2.2 | -24.4 |

**Table S4**. Affinity energy in previously reported ToBRFV MP mutants.

| **Amino acid substitution** | **ΔΔG (kcal mol^-1^)** | | **Interface** |
| --- | --- | --- | --- |
|  | **Tm-2^2^ /ToBRFV MP** | **ToBRFV MP/Tm-2^2^** |  |
| H67C | -0.25 | -0.08 | Yes |
| N125A | 0.60 | 0.25 | Yes |
| K129Q | 0.17 | 0.32 | No |
| A134N | -1.02 | -0.65 | No |
| I147M | -0.28 | -0.05 | No |
| I168N | -0.70 | -0.58 | Yes |
